# *EGFR, KRAS, BRAF*, and *HER-2* molecular status in brain metastases from 77 NSCLC patients

**DOI:** 10.1002/cam4.82

**Published:** 2013-04-23

**Authors:** Claire Villalva, Valérie Duranton-Tanneur, Karline Guilloteau, Fanny Burel-Vandenbos, Michel Wager, Jérôme Doyen, Pierre Marie Levillain, Denys Fontaine, Hélène Blons, Florence Pedeutour, Lucie Karayan-Tapon

**Affiliations:** 1 INSERM U935, Poitiers UniversityPoitiers, France; 2 Laboratory of Molecular Oncology, Poitiers University HospitalPoitiers, France; 3 Laboratory of Solid Tumors Genetics, Faculty of Medicine, Nice University HospitalNice, France; 4 Central Department of Pathology, Faculty of Medicine, Nice University HospitalNice, France; 5 Department of Neurosurgery, Faculty of Medicine, Poitiers University HospitalPoitiers, France; 6 Department of Pathology, Poitiers University HospitalPoitiers, France; 7 Department of Neurosurgery, Faculty of Medicine, Nice University HospitalNice, France; 8 Departement of Biology, European Georges Pompidou Hospital, AP-HPParis, France; 9 INSERMU775, Paris Descartes UniversityParis, France; 10 Institute of Research on Cancer and Ageing in Nice (IRCAN), Sophia-Antipolis UniversityNice, France

**Keywords:** *BRAF*, brain metastases, epidermal growth factor receptor, *HER-2*, *KRAS*, non-small cell lung cancer

## Abstract

The aim of this study was to determine the frequency of *EGFR, KRAS*, *BRAF,* and *HER-*2 mutations in brain metastases from non-small cell lung carcinomas (BM-NSCLC). A total of 77 samples of BM-NSCLC were included and 19 samples of BM from breast, kidney, and colorectal tumors were also studied as controls. These samples were collected from patients followed between 2008 and 2011 at Poitiers and Nice University Hospitals in France. The frequencies of *EGFR*, *KRAS*, *BRAF,* and *HER-*2 mutations in BM-NSCLC were 2.6, 38.5, 0, and 0% respectively. The incidence of *KRAS* mutation was significantly higher in female and younger patients (*P* < 0.05). No mutations of the four genes were found in BM from breast or kidney. However, among six BM from colorectal tumors, we identified *KRAS* mutations in three cases and *BRAF* mutations in two other cases. This study is the largest analysis on genetic alterations in BM-NSCLC performed to date. Our results suggest a low frequency of *EGFR* mutations in BM-NSCLC whereas *KRAS* mutations are as frequent in BM-NSCLC as in primitive NSCLC. These results raise the question of the variability of the brain metastatic potential of NSCLC cells in relation to the mutation pattern.

## Introduction

Among all malignant tumors, non-small cell lung cancer (NSCLC) is the main cause of brain metastases (BM). BM arise in approximately 20–40% of NSCLC patients [1]. They are mainly detected synchronously to NSCLC (50% of cases), but can also be prevalent (6% of cases) or metachronous [2]. They are symptomatic in 85% of patients. The prognosis is generally poor, with a median survival of 4–11 weeks in untreated patients, but it is improved in patients treated by whole-brain radiation therapy (WBRT), with a median survival of 3–6 months [3]. Surgery and stereotactic radiosurgery are therapeutic options for oligometastatic disease and must be considered when possible. Combined with WBRT, surgery or radiosurgery improves the overall survival rate, with a median of 6–11 months [4, 5]. WBRT remains the standard therapy for multiple brain metastases, but NSCLC is a radioresistant cancer and 30 Gy WBRT is not sufficient to sterilize the lesions. Treatment of BM by chemotherapy remains controversial, since uncertain penetration of anti-cancer drugs through the blood–brain barrier restrains their optimal use. Several targeted therapies have recently been developed in the treatment of NSCLC, the efficiency of which depends on predictive value of molecular biomarkers' mutational status. Indeed, approximately 60–80% of patients whose tumor samples contain somatic mutations in the kinase domain of *epidermal growth factor receptor (EGFR)* gene are responsive to *EGFR* tyrosine kinase inhibitors (TKI) gefitinib and erlotinib [6]. More than 80% of the detected mutations are located at amino acids 746–753 encoded by exon 19 and amino acid 858 encoded by exon 21 [7]. KRAS protein, other downstream effectors of EGFR such as serine-threonine kinase BRAF, and another member of the human epidermal growth factor receptor (HER) family, *v-erb-b2 erythroblastic leukemia viral oncogene homolog 2* (*HER-2*), are also implicated in the tumorigenesis and progression of NSCLC [8, 9]. Approximately 97% of *KRAS* mutations in primary NSCLC involve codons 12 or 13. The most frequent *BRAF* and *HER-2* mutations in NSCLC are amino acidic substitution of p.V600E in exon 15 and a 12-bp duplication coding for the amino acids YVMA at codon 776, respectively [10, 11]. Although the impact of these mutations has not been completely elucidated, recent publications have shown that they represent negative prognostic markers in NSCLC [12, 13]. While the predictive value of wild-type *KRAS* genotype for identifying patients who will benefit from anti-EGFR monoclonal antibodies treatment is now well established in metastatic colorectal cancer, the significance of *KRAS* and *BRAF* mutations in NSCLC is not yet comparably clear [14–17]. The potential effectiveness of BRAF, HER-2, MEK, and mTOR inhibitors in the presence of mutations is currently being investigated in clinical trials [18, 19]. While the molecular status of *EGFR* in primary NSCLC has been widely studied, data concerning the molecular status of BM from NSCLC are scarce [20–26]. However, it is known that BM from NSCLC responds to oral EGFR TKIs according to the presence of activating mutations [6, 27, 28]. Recently, studies about the molecular pathways that mediate brain colonization have shown that genetic factors play an important role and that the molecular status of oncogenes is part of the risk-stratification of patients and needs to be investigated [29].

In this article we present to the best of our knowledge, a molecular study with clinical data of the largest series of BM from NSCLC (BM-NSCLC). The aim was to investigate the frequencies of *EGFR*, *KRAS*, *BRAF,* and *HER-2* mutations in BM-NSCLC samples from 77 patients operated in the neurosurgery departments of Nice and Poitiers University Hospitals (France). In addition, we established and compared the mutational status of eight pairs of primary NSCLC and matched BM-NSCLC and examined the frequencies of the same mutations in 19 BM from tumors other than NSCLC, such as breast, kidney, and colorectal cancers.

## Materials and Methods

### Samples

Formalin-fixed and paraffin-embedded BM tumor samples from fine needle aspiration or surgical resection were obtained from 96 patients, mainly of Caucasian origin, treated between 2008 and 2011 at Poitiers and Nice Hospitals in France. The histological types were as follows: Stage IV NSCLC *n* = 77, breast *n* = 7, kidney *n* = 6, and colorectal *n* = 6. None of the patients had previously been treated with EGFR inhibitors. The frequencies of *EGFR, KRAS*, *BRAF,* and *HER-2* mutations in BM-NSCLC from our series were compared to frequencies of these mutations in a cohort of stage IV-primitive NSCLC samples evaluated for *EGFR* (*n* = 1235)*, KRAS* (*n* = 1046), *BRAF* (*n* = 734), and *HER-2* (*n* = 284) in our molecular diagnosis daily practice between 2009 and 2012, according to the recommendations of the French National Cancer Institute (INCa) (http://www.e-cancer.fr). The samples had been collected after informed consent of all patients according to the ethical rules of our institutions.

### Genetic analysis

The presence of at least 50% tumor cells in samples was evaluated histologically. Genomic DNA was extracted using DNAeasy Blood & Tissue DNA isolation kit or QIAamp DNA Mini Kit (Qiagen, Hilden, Germany). Genotyping of *EGFR* exons 18, 19, 20, and 21 was performed by pyrosequencing method with PyroMark Q24 and CE-IVD-marked Therascreen EGFR Pyro associated Kit (Qiagen) or length analysis of fluorescently labelled PCR products for exons 19 and 21. To reveal the presence of p.L858R, PCR product of exon 21 was digested by Sau96I (New England Biolabs, Evry, France). Genotyping was carried out using 3500 Genetic Analyzer (Applied Biosystems, Foster City, CA) and results were interpreted using Genemapper V4.1 software (Applied Biosystems).

Genotyping of *KRAS* exon 2 (codons 12 and 13) was performed by pyrosequencing method with the PyroMark Q24 and CE-IVD-marked Therascreen KRAS Pyro associated kit (Qiagen) or allelic discrimination using the 7500 Fast Real-Time PCR platform (Applied Biosystems). The design of sequences of the TaqMan probes was kindly provided by Pr Laurent-Puig [30].

Genotyping of *BRAF* exon 15 and *HER-2* exon 20 was performed by pyrosequencing method with sequences designed using PyroMark Assay Design Software (Qiagen). Pyrosequencing was done according to the manufacturer's instructions (Qiagen). Results were interpreted using PyroMark Q24 2.0 software (Qiagen). The primers sequences and PCR conditions are available on request.

### Statistical methods

Statistical analyses were performed to characterize the relationships between mutational status and clinical features by using chi square or Mann–Whitney tests. The statistical analyses were two-tailed ones and the level of significance was set at *P* = 0.05.

## Results and Discussion

### Clinical characteristics of patients

Seventy-seven consecutive cases of BM from NSCLC non-treated with TKI were studied (Table 1). The histological types were adenocarcinomas in 71 cases, squamous cell carcinomas in five cases, and large cell carcinoma in one case. All patients initially presented a stage IV disease. The mean age was 59.2 ± 10.1 years. A male predominance of 69.2% was observed (male-to-female ratio = 1:0.44). Sixty-six tumors were located in the supratentorial compartment while seven were cerebellar metastases. In addition, the clinical characteristics of 19 patients affected by BM from diverse primary tumors other than NSCLC are described in Table 2.

**Table 1:** *EGFR, KRAS, BRAF, HER-2, and ALK* mutation status in brain metastases from NSCLC and patient characteristics

			Mutation status in brain metastases						
					
No	Sex	Age	*EGFR*	*KRAS*	*BRAT*	*HER-2*	*ALK*	Metastases anatomical location	Smoking status
BM1	F	70	wt	G12D	wt	wt	nc	Supratentorial	nc
BM2	M	40	wt	G12C	wt	wt	nc	Supratentorial	Current smoker
BM3	M	60	wt	wt	wt	wt	nc	Supratentorial	nc
BM4	F	47	wt	wt	wt	wt	nc	Supratentorial	Ex-smoker
BM5	M	54	wt	wt	wt	wt	nc	nc	nc
BM6	F	50	wt	G12C	wt	wt	nc	Supratentorial	nc
BM7	M	66	wt	wt	wt	wt	nc	Infratentorial	nc
BM8	M	65	wt	G12C	wt	wt	nc	Supratentorial	nc
BM9	F	75	L858R	wt	wt	wt	nc	Supratentorial	nc
BM10	M	69	wt	wt	wt	wt	nc	Supratentorial	Current smoker
BM11	F	57	wt	G12V	wt	nc	nc	Supratentorial	nc
BM12	M	80	wt	wt	wt	wt	nc	Supratentorial	Ex-smoker
BM13	M	56	L858R	wt	wt	wt	nc	Supratentorial	Non smoker
BM14	M	54	wt	wt	wt	wt	nc	Supratentorial	Current smoker
BM15	F	54	wt	G12C	wt	wt	nc	Supratentorial	nc
BM16	M	54	wt	G12V	wt	wt	nc	Supratentorial	Current smoker
BM17	M	54	wt	G12C	wt	wt	nc	nc	Current smoker
BM18	M	54	wt	wt	wt	wt	No rearrangement	Supratentorial	nc
BM19	M	64	wt	G12V	wt	wt	nc	nc	nc
BM20	M	67	L858R	wt	wt	wt	nc	Infratentorial	nc
BM21	M	57	wt	wt	wt	wt	nc	Supratentorial	Current smoker
BM22	M	77	wt	wt	wt	wt	No rearrangement	Supratentorial	Ex-smoker
BM23	M	66	wt	G12C	wt	wt	nc	Supratentorial	nc
BM24	M	55	wt	wt	wt	wt	nc	Supratentorial	Ex-smoker
BM25	F	60	wt	G12V	wt	wt	nc	Supratentorial	nc
BM26	F	59	wt	G12D	wt	wt	nc	Supratentorial	Current smoker
BM27	M	79	wt	G12V	wt	wt	nc	Supratentorial	Ex-smoker
BM28	M	57	wt	wt	wt	wt	nc	Supratentorial	nc
BM29	M	59	wt	wt	wt	wt	No rearrangement	nc	nc
BM30	M	61	wt	wt	wt	wt	nc	Supratentorial	nc
BM31	F	52	wt	G13D	wt	wt	nc	Supratentorial	nc
BM32	M	71	wt	wt	wt	wt	nc	Supratentorial	Current smoker
BM33	M	58	wt	wt	wt	wt	nc	Infratentorial	Current smoker
BM34	M	83	wt	wt	wt	wt	nc	Supratentorial	nc
BM35	M	61	wt	G12C	wt	wt	nc	Supratentorial	Current smoker
BM36	M	66	wt	wt	wt	wt	nc	Supratentorial	Current smoker
BM37	F	54	wt	wt	wt	wt	nc	Supratentorial	Current smoker
BM38	M	60	wt	wt	wt	wt	nc	Supratentorial	Ex-smoker
BM39	F	42	wt	G12C	wt	wt	nc	Supratentorial	nc
BM40	F	52	wt	wt	wt	wt	No rearrangement	Supratentorial	Current smoker
BM41	M	54	wt	G12F	wt	wt	nc	Supratentorial	Ex-smoker
BM42	F	68	wt	G12A	wt	wt	nc	Supratentorial	Ex-smoker
BM43	M	52	wt	G12V	wt	wt	nc	Supratentorial	Non smoker
BM44	M	52	wt	G12V	wt	wt	nc	Supratentorial	Current smoker
BM45	M	52	wt	G12C	wt	wt	nc	Supratentorial	Current smoker
BM46	F	58	wt	G12C	wt	wt	nc	Supratentorial	Ex-smoker
BM47	M	61	wt	wt	wt	wt	nc	Supratentorial	Current smoker
BM48	M	67	wt	wt	wt	wt	nc	Supratentorial	Current smoker
BM49	M	58	wt	wt	wt	wt	nc	Supratentorial	Current smoker
BM50	F	57	wt	wt	wt	wt	nc	Supratentorial	nc
BM51	F	65	wt	wt	wt	wt	nc	Supratentorial	Ex-smoker
BM52	M	59	wt	wt	wt	wt	nc	Supratentorial	Current smoker
BM53	M	81	wt	wt	wt	wt	nc	Supratentorial	Ex-smoker
BM54	M	70	wt	wt	wt	wt	nc	Supratentorial	Ex-smoker
BM55	M	65	wt	wt	wt	wt	nc	Supratentorial	Current smoker
BM56	M	66	wt	wt	wt	wt	nc	Supratentorial	nc
BM57	F	49	wt	wt	wt	wt	nc	Supratentorial	Current smoker
BM58	M	69	wt	wt	wt	wt	nc	Supratentorial	nc
BM59	M	67	wt	wt	wt	wt	nc	Infratentorial	Current smoker
BM60	F	35	wt	wt	wt	wt	nc	Infratentorial	nc
BM61	M	58	wt	wt	wt	wt	nc	Supratentorial	Ex-smoker
BM62	M	39	wt	wt	wt	wt	nc	Supratentorial	nc
BM63	F	48	wt	wt	wt	wt	nc	Supratentorial	Non smoker
BM64	M	53	wt	G13D	wt	wt	nc	Supratentorial	Ex-smoker
BM65	M	80	wt	G12V	wt	wt	nc	Supratentorial	Current smoker
BM66	M	52	wt	wt	wt	wt	nc	Supratentorial	Ex-smoker
BM67	M	64	wt	wt	wt	wt	nc	Supratentorial	Current smoker
BM68	M	55	wt	wt	wt	wt	No rearrangement	Supratentorial	Ex-smoker
BM69	F	45	wt	G12C	wt	wt	No rearrangement	Supratentorial	Current smoker
BM70	F	47	wt	G12V	wt	wt	No rearrangement	Supratentorial	nc
BM71	F	53	wt	G12C	wt	wt	No rearrangement	Supratentorial	nc
BM72	F	47	wt	G12V	wt	wt	No rearrangement	Supratentorial	nc
BM73	M	57	wt	G12A	wt	wt	No rearrangement	Supratentorial	nc
BM74	M	63	wt	wt	wt	wt	No rearrangement	Supratentorial	nc
BM75	M	59	wt	wt	wt	wt	No rearrangement	Supratentorial	nc
BM76	M	75	wt	wt	wt	wt	No rearrangement	Supratentorial	nc
BM77	F	43	wt	G12V	wt	wt	No rearrangement	Supratentorial	nc

BM, brain metastase; wt, wild-type; ADC, adenocarcinoma; SCC, squamous cell carcinoma; LCC, large cell carcinoma; nc, noncommunicated.

**Table 2:** *EGFR*, *KRAS*, *BRAF*, and *HER-2* mutation status in brain metastases from breast, kidney, and colorectal primitive tumors and patient characteristics

				Mutation status in brain metastases			
				
Primitive anatomical location	No	Sex	Age	*EGFR*	*KRAS*	*BRAF*	*HER-2*
Breast	BM70	F	79	wt	wt	wt	wt
	BM71	F	63	wt	wt	wt	wt
	BM72	F	63	wt	wt	wt	wt
	BM73	F	71	wt	wt	wt	wt
	BM74	F	40	wt	wt	wt	wt
	BM75	F	37	wt	wt	wt	wt
	BM76	F	60	wt	wt	wt	wt
Kidney	BM77	M	50	wt	wt	wt	wt
	BM78	M	67	wt	wt	wt	wt
	BM79	F	69	wt	wt	wt	wt
	BM80	F	57	wt	wt	wt	wt
	BM81	M	64	wt	wt	wt	wt
	BM82	F	57	wt	wt	wt	wt
Colorectal	BM83	F	77	wt	wt	wt	wt
	BM84	M	67	wt	wt	V600E	wt
	BM85	F	80	wt	G13D	wt	wt
	BM86	M	58	wt	wt	V600E	wt
	BM87	M	59	wt	G12A	wt	wt
	BM88	M	76	wt	G12V	wt	wt

### *EGFR* mutations in brain metastases from NSCLC and in matched primitive NSCLC

We detected p.L858R mutation of *EGFR* in three out of 77 cases of BM-NSCLC (3.9%). One patient was a non-smoker male while the smoking status of the other two patients (one male and one female) was not known. No mutation of *KRAS, BRAF,* and *HER-2* was detected in these three *EGFR*-mutated samples (Table 1). Recent studies have shown that the EGFR TKI are active in patients with BM-NSCLC but unfortunately, none of the three patients in our series had received this treatment [27, 28]. The frequency of 3.9% of *EGFR* mutations in our series of BM-NSCLC is lower than rates in grouped series of primary tumors and all anatomically located metastases. Indeed, the frequency of *EGFR* mutation in published series of NSCLC is approximately 10–16% in patients of non-Asian origin [31, 32]. In France, according to the INCa register, frequency of *EGFR*-activating mutation was 10% in 20,750 NSCLC patients tested in 2011 (http://www.e-cancer.fr). In our own experience of daily practice in Nice and Poitiers between 2009 and 2012, while using the same experimental methods as those described here, we observed a mutation frequency of 9.5% (1235 NSCLC patients). The ethnic origins of patients are determining factors since frequencies of *EGFR* mutation are much higher (40%) in patients of Asian origin [33]. The difference between frequency of 3.9% in BM-NSCLC versus 9.5 or 10% in NSCLC is statistically significant (*P* < 0.05) and this low frequency of *EGFR* mutations in BM is consistent with results from other studies in Caucasian patients. Sun et al. [34], Daniele et al. [21], and Cortot et al. [20] reported frequencies of 1/42, 0/28, and 0/13 of *EGFR* mutations in BM-NSCLC, respectively. Several hypotheses can be raised in explanation of this low frequency in BM-NSCLC. First, the low frequency of *EGFR* mutations could be explained by the male gender, smoker and non-Asian predominance in our BM-NSCLC series since it has been shown that *EGFR* mutations in NSCLC are statistically associated with female gender, non-smoking status, and Asian origin [35, 36]. Male and Caucasian predominance is also present in the three studies mentioned above. Smoking status was not mentioned. As a second hypothesis, it should be considered that tumor heterogeneity at the molecular level might be responsible for the differences in frequency between primitive-NSCLC and BM-NSCLC. We could study only eight matched cases and found no discrepancy of *EGFR* status in primitive tumors and matched BM (Table 3). Other studies have reported heterogeneity of *EGFR* status, but a recent article in a large Asian series explains these discordances by technical art**e**facts due to heterogeneity in the amplification of *EGFR* mutated [22, 23, 26, 37]. In the studies of Sun et al. and Cortot et al. [20, 34], all cases showed concordant results between NSCLC primary tumors and BM. The report by Daniele et al. [21] only covers mutation status in BM. Finally, as a third hypothesis, one may think that *EGFR* mutational status impacts the capacity to metastasize. Doebele et al. [10] have shown that the mutation profile (*EGFR/KRAS/ALK*) did impact the metastatic spread pattern and one can hypothesize that wild-type *EGFR* clones have enhanced brain metastatic potential and that patients with *EGFR*-mutated tumors may develop fewer BM than patients with wild-type *EGFR* tumors. Consistent with this hypothesis, two recent studies have reported a longer median time to progression for patients with BM harboring *EGFR* mutations in their primitive NSCLC compared to patients whose *EGFR* mutational status was not known or wild type [27, 38]. Another study has suggested a lower risk of CNS invasion in patients with advanced *EGFR*-mutated NSCLC treated with initial systemic therapy by gefitinib or erlotinib than the risk reported in historical series (19% vs. 40%) [39]. However, these results may be dependent on EGFR-TKI treatment rather than *EGFR* status by itself. On the other hand, the study of Li et al. [40] has suggested that BM would be more frequent in patients with tumors bearing *EGFR* mutations. In this retrospective study including 110 patients with NSCLC, *EGFR* status was determined in primitive tumors and compared with the development of BM. The frequencies of *EGFR* mutation were 64% and 31% in the patients with and without BM, respectively. Thus, current published data are far too limited to draw any firm conclusion and it is necessary to study large case series.

**Table 3:** *EGFR*, *KRAS*, *BRAF, and HER-2* mutation status in paired primitive NSCLC and BM samples

	Mutation status in primitive NSCLC				Mutation status in brain metastases			
		
	*EGFR*	*KRAS*	*BRAF*	*HER-2*	*EGFR*	*KRAS*	BRAF	*HER-2*
BM3	wt	wt	wt	wt	wt	wt	wt	wt
BM9	L858R	wt	wt	wt	L858R	wt	wt	wt
BM13	L858R	wt	wt	wt	L858R	wt	wt	wt
BM20	L858R	wt	wt	wt	L858R	wt	wt	wt
BM22	wt	wt	wt	wt	wt	wt	wt	wt
BM26	wt	G13D	wt	wt	wt	G13D	wt	wt
BM35	wt	G12C	wt	wt	wt	G12C	wt	wt
BM43	wt	G12V	wt	wt	wt	G12V	wt	wt

### *KRAS* mutations in brain metastases from NSCLC and in matched primitive NSCLC

We have detected mutations of *KRAS* in 30 out of 77 (39.0%) BM-NSCLC cases (Table 1). Among them, mutations in codon 12 were observed in 26 cases and in codon 13 in four cases (Table 4). Little information concerning the brain localization is available in the few reports on *KRAS* status in lung cancer metastases [20, 25, 26, 41, 42]. To note, the frequency of 39.0% is not statistically different from the frequency of 31.3% that we observed in 1046 primary NSCLC between 2009 and 2012 using the same technology as for the BM-NSCLC. According to the INCa register, the frequency of *KRAS* mutation was 25.4% in 17,153 patients (primary and all metastatic sites) tested in 2011 (http://www.e-cancer.fr). This frequency is lower, but the use of various techniques with lower sensitivities than pyrosequencing and allelic discrimination can explain this difference. In our cohort of patients with BM-NSCLC, the incidence of *KRAS* mutation was significantly higher in female than in male patients and in younger ones (*P* < 0.05) (Table 5). These points have already been reported in several studies about NSCLC and the sex-linked factors that are related to lung cancer risk deserve consideration [43, 44]. No association of frequency of *KRAS* mutations with tumor differentiation was found. Correlation of *KRAS* mutation with smoking history has previously been reported [45]. In our study, smoking status was known in 44 cases, and only three patients were nonsmokers, including one case with *KRAS* mutation. No correlation was found between *KRAS* mutations and smoking status. Interestingly, we have found no *KRAS* mutation when the metastases were located in the cerebellum (Table 5). As for EGFR mutations, we did not observe discordant results between primitive and metastatic tumors for *KRAS* mutations, indicating that they are generally acquired prior to metastatic spread, but our matched series and studies on this subject are still too few to draw firm conclusions (Table 3). As we found comparable frequencies of *KRAS* mutations in primitive NSCLC and in BM, our data are consistent with the idea that *KRAS* mutational status does not influence the capacity of cells from NSCLC to metastasize. In NSCLC, several studies showed that *KRAS* mutations were associated with decreased time to progression and shorter survival [12, 16]. mTOR and MEK inhibitors are currently being evaluated in clinical trials in patients with *KRAS* mutations [18, 19]. In BM-NSCLC, further investigations are needed to evaluate the efficacy of these inhibitors.

**Table 4:** Summary of *KRAS* mutations in brain metastases from NSCLC (*n* = 30)

*KRAS*	Nucleotide change	Codon change	Cases
*Codon 12*	GGT>GAT	G12D	1 (3.3%)
	GGT>TGT	G12C	11 (36.7%)
	GGT>GTT	G12V	11 (36.7%)
	GGT>GCT	G12A	2 (6.6%)
	GGT>TTT	G12F	1 (3.3%)
*Codon 13*	GGOGAC	G13D	3 (9.9%)
	GGOTGC	G13C	1 (3.3%)

**Table 5:** Correlations between *KRAS* status and clinicopathologic factors in BM from NSCLC patients

		Gender		Histopathology			Anatomical location		Smoking status		
					
*KRAS* mutation status	Age (mean)	Male	Female	Poorly differentiated	Moderately differentiated	Well differentiated	Infratentorial	Supratentorial	Non-smoker	Ex-smoker	Current smoker
Wild-type	61.1 ± 10.0	38	9	10	11	3	7	24	1	10	16
Mutated	56.3 ± 9.8	15	15	7	6	1	0	24	1	4	9
Statistical significativity	*P* < 0.05	*χ*^2^ = 8.125 *P* < 0.05			*χ*^2^ = 0.396 *P* > 0.05		*χ*^2^ = 6.21 *P* < 0.05		*χ*^2^ = 0. 454 *P* > 0.05		

### *BRAF* and *HER-2* mutations in brain metastases from NSCLC

**Figure 1:**
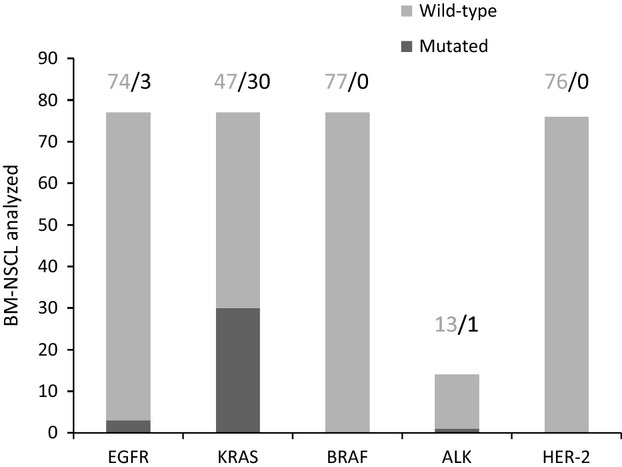
Spectrum of analyzed mutations in our series of BM-NSCLC.

No mutations of *BRAF* or *HER-2* were found, respectively, in 77 and 76 analyzed cases of BM-NSCLC (Table 1). To our knowledge, this is the first study to have investigated *BRAF* and *HER-2* mutational status in BM-NSCLC. Concerning primary-NSCLC and all metastatic sites analyzed in 2011, the INCa register indicates *BRAF* and *HER-2* mutation frequencies of 1.8% in 10,017 patients and 0.9% in 7731 patients, respectively (http://www.e-cancer.fr). Our daily practice between 2009 and 2012, with the same technology shows *BRAF* and *HER-2* mutation frequencies of 2.5% (734 patients) and 1.4% (284 patients), respectively. Genetic rearrangement between *echinoderm microtubule-associated protein-like 4 and anaplastic lymphoma kinase* (*EML4-ALK*) was examined in 14 BM-NSCLC and one was positive. Although this genetic alteration is usually described as exclusive with *EGFR* or *KRAS* mutations, in our series, this patient also harbors *KRAS* mutation (BM73). The spectrum of the five driver mutations in our series of BM-NSCLC is illustrated in Figure 1.

### *EGFR, KRAS, BRAF*, and *HER-2* mutations in BM from primitive tumors other than lung adenocarcinomas

Frequencies of *EGFR, KRAS*, *BRAF*, and *HER-2* mutations were compared between BM from lung, breast, kidney, and colorectal tumors (Table 2). All metastases studied were wild type for *EGFR*. No *KRAS* mutations were found in metastases from breast and kidney whereas three of six metastases from colorectal tumors were mutated. Despite the small size of the series, the results suggest that BM from lung and colorectal tumors are more frequently *KRAS*-mutated than those from breast and kidney. Indeed, we have noticed statistical differences in frequency of *KRAS* mutations between BM from NSCLC and from breast and kidney (*P* < 0.05), but no difference between NSCLC and colorectal tumor (*P* > 0.05) (Table 6). A high frequency of mutations in BM from colorectal cancers has been reported by Tie et al. [46]. Two cases of BM from colorectal tumors were *BRAF* mutated (p.V600E). A similar observation was made in BM from melanoma by El-Osta et al. [47] that showed more BM in melanoma patients with mutated *BRAF* versus wild type. No mutation of *HER-2* was found in all cases of metastases.

**Table 6:** Correlations between frequency of *KRAS* mutations in BM from breast, kidney, or colorectal tumors and BM from NSCLC

	BM from breast tumors	BM from kidney tumors	BM from colorectal tumors
BM-NSCLC	*χ*^2^ = 4.988 *P* < 0.05	*χ*^2^ = 4.331 *P* < 0.05	*χ*^2^ = 0.072 *P*> 0.05

In summary, we report the first large series analyzing *EGFR, KRAS*, *BRAF,* and *HER-2* mutations in brain metastases of NSCLC. While the frequencies of *KRAS* and *BRAF* mutations were similar to frequencies usually described in primitive or other metastatic locations of NSCLC, the frequency of EGFR mutations was low. Mechanistic studies are needed to evaluate the association of these mutations with the metastatic spread. We can assume that they are not necessary to trigger the metastatic process since the majority of patients with metastases do not have these mutations. BM-NSCLC have been shown to respond to oral EGFR TKIs and these data highlight the potential value of detecting mutations for choosing the most appropriate targeted treatment and for surveillance strategies.
